# Effect of Oxygen-deficiencies on Resistance Switching in Amorphous YFe_0.5_Cr_0.5_O_3−d_ films

**DOI:** 10.1038/srep30335

**Published:** 2016-07-25

**Authors:** Xianjie Wang, Chang Hu, Yongli Song, Xiaofeng Zhao, Lingli Zhang, Zhe Lv, Yang Wang, Zhiguo Liu, Yi Wang, Yu Zhang, Yu Sui, Bo Song

**Affiliations:** 1 Department of Physics, Harbin Institute of Technology, Harbin 150001, China; 2 Academy of Fundamental and Interdisciplinary Sciences, Harbin Institute of Technology, Harbin 150001, China

## Abstract

Herein, we demonstrate the contribution of the oxygen-deficiencies on the bipolar resistance switching (RS) properties of amorphous-YFe_0.5_Cr_0.5_O_3−d_ (a-YFCO) films. The a-YFCO films were prepared under various oxygen pressures to tune the concentration of oxygen-deficiencies in the films. The XPS data verify that the oxygen-deficiencies increase with decreasing oxygen pressure. The RS property becomes more pronounced with more oxygen-deficiencies in a-YFCO films. Based on the Ohmic conduction measurements in the low resistance state, we confirm that the RS mechanism is related to the migration of oxygen-deficiencies. The enhanced RS and long retention in a-YFCO suggest a great potential for applications in nonvolatile memory devices.

Resistance switching (RS) has been observed in metal/RS layer/metal structures, in which the resistance can be switched reversibly between a high resistance state (HRS) and a low resistance state (LRS) depending on the applied voltage^1^,^2^,^3^. These two resistance states can be applied in resistance random access memory (ReRAM) devices, which have been investigated widely as promising candidates for commercial nonvolatile memory due to their low power consumption, fast switching speed, and scalability^4^,^5^,^6^. Furthermore, some studies demonstrate that the actual active switching region occurs in a nano-scale region between metal electrodes and RS layers, which suggests great potential applications for nonvolatile memory device^2^,^7^. Until now, a variety of materials had been investigated as RS layers in ReRAM devices, such as transition metal (TM) oxides^8^,^9^,^10^, ferroelectric films^11^,^12^,^13^, and perovskite oxides^14^,^15^,^16^. Many models have been proposed to explain the RS behaviors, such as conductive filament (CF) model^17^,^18^,^19^, Schottky barrier model^20^, and space-charge limited current model^21^. A common perspective is that the migration of oxygen vacancies (V_O_s) plays a dominant role on the RS for oxides. And, the formation or rupture of the CF is due to the interconnected or disconnected oxygen-deficiencies at various applied voltages, respectively^22^,^23^,^24^,^25^,^26^,^27^,^28^,^29^,^30^. Recently, Xu *et al*. mapped out the V_O_s directly and demonstrated that V_O_s are responsible for RS in LaMnO_3−x_ films^15^. Then, Li *et al*. reported a direct observation of the dynamic process of V_O_s migration in CeO_2_ film driven by electric field^31^. Both of these previous investigations clearly indicate that V_O_s are in fact uniformly distributed in the RS layer, and a large amount of V_O_s in oxide film themselves merely serves as a V_O_s reservoir, that is, the performance of RS is strongly affected by the concentrations of V_O_s.

More recently, large RS ratio and long retention could be achieved in amorphous-film-based ReRAM devices, and suggested that it is very attractive for future applications of nonvolatile ReRAM devices^32^,^33^,^34^. In contrast to the polycrystalline material, amorphous material has a structural homogeneity on the atomic scale, which is beneficial to the ionic migration and then enhances the performance of ReRAM. However, each step must be carefully controlled to avoid any heating or chemical damaging/etching effect on the amorphous film to prevent crystallization during the entire fabrication process of the devices because the amorphous films was prepared at low temperature. Therefore, the amorphous film that can be prepared at high temperature should beneficial to the fabrication of the RS devices and thus its’ applications^35^,^36^,^37^,^38^. YFe_0.5_Cr_0.5_O_3_ (YFCO) is a perovskite oxide with an orthorhombic structure^39^. The Fe and Cr cations are randomly positioned instead of the 1:1 ordering in the *B* sub-lattice, and its crystallization temperature is as high as 1150 °C. The YFCO slice has multiferroic properties at room temperature, which can offer additional degrees of freedom for YFCO-based multifunctional devices^40^. But there is no experimental work of YFCO film with a stable crystalline structure due to the growth difficulty of the YFCO films. However, the amorphous YFCO film with high crystallization temperature should beneficial to the fabrication of the RS devices. In this paper, we investigate for the first time the RS properties of amorphous-YFCO (a-YFCO) films prepared under various oxygen pressures and high temperature. A bipolar RS with a large resistance ratio at room temperature was observed. The enhanced RS and long retention suggest its great potential for applications in nonvolatile memory devices.

## Results and Discussions

**Figure 1:**
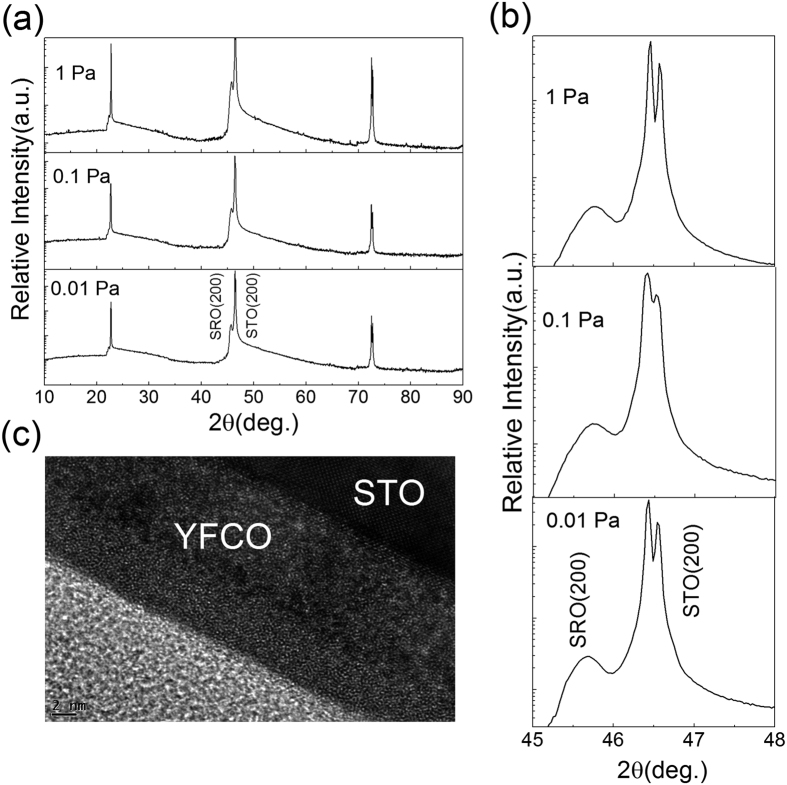
(**a**) XRD patterns of the a-YFCO/SRO/STO structure fabricated under different oxygen pressures. (**b**) (200) peaks of SRO and STO. (**c**) The high-resolution transmission electron microscopy (HRTEM) image of a-YFCO/SRO/STO.

Figure 1(a) shows the XRD patterns for the YFCO/SrRuO_3_(SRO)/ SrTiO_3_(STO) structure. Only the reflections from the (001) family of STO and SRO were observed, indicating that a single-phase SRO epitaxial film was formed. No obvious peaks associated with YFCO films were observed because of the amorphous crystal structure of the as-synthesized a-YFCO films. The high-resolution transmission electron microscopy (HRTEM) micrographs give a clear view of the YFCO/STO structure, which clearly suggested that the YFCO film is amorphous, as shown in Fig. 1(b). It is well known that the deposition parameters, especially the oxygen pressure, can significantly affect the microstructure of the oxides^15^. Figure 1(b) shows the (002) peaks of SRO in more detail. The diffraction peaks of the SRO films shift a little towards lower diffraction angles with a decrease of the oxygen pressure, due to the formation of oxygen-deficiencies at different oxygen pressures, which can be considered as a partial V_O_s reservoir in the RS properties as suggested by Moors *et al*.^8^.

**Figure 2:**
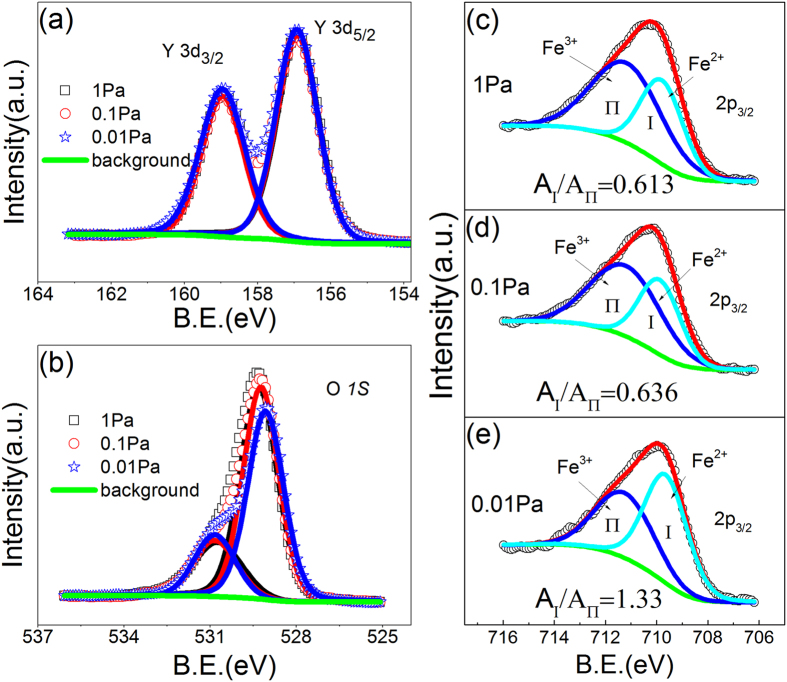
XPS data of the elements Y (**a**), O (**b**) and Fe (**c**–**e**) in the a-YFCO films grown under different oxygen pressures.

We measured the XPS of the surface of a-YFCO films in order to avoid the removal of oxygen atoms and subsequent oxide reduction during the depth profile argon milling process. The *ex-situ* transferred measurement may introduce extra oxygen because of the atmosphere, but the ex-site XPS had been used widely in oxides to identify the corresponding chemical valence states and the V_O_s and oxygen-deficiencies^8^,^41^,^42^. Thus the ex-site XPS is useful to identify the oxygen ion concentration in a-YFCO films, here the C peak was used to align the energy axis for each sample. Two strong peaks at 159.35 and 156.85 eV correspond to Y 3d_3/2_ and Y 3d_5/2_, respectively. Obviously, the oxygen pressure has no impact on the valence and concentration of Y element, as shown in Fig. 2(a). But the O1s spectra is de-convoluted into two Gaussian–Lorentzian curves located at 529.2 ± 0.1 and 530.8 ± 0.2 eV, respectively, as shown in Fig. 2(b). The main peak at 529.2 ± 0.1 eV is attributed to oxygen in a-YFCO, such as Fe-O, Cr-O and Y-O, which is strongly associated with oxygen-deficiencies, and the peak at 530.8 ± 0.2 eV should be attributed to surface oxygen that is chemisorbed or bound electrostatically on the surface of YFCO^41^,^42^,^43^,^44^,^45^. With decreasing oxygen pressure, the integral area of O at 529.2 ± 0.1 eV gradually decreases but the integral area of Y changes little, which indicates a decrease of oxygen ion concentration in the a-YFCO films, as shown in Fig. 2(b). The main peak at 529.2 ± 0.1 eV shifts to lower binding energies with decreasing oxygen pressure because of the increasing density of electronic states induced by oxygen-deficiencies, which further confirm the oxygen ion concentration decrease with decreasing oxygen pressure^46^. The Fe 2p XPS data in the a-YFCO films are shown in the Fig. 2(c–e) and S1 of Supporting Information. Clear signals of Fe^3+^ and Fe^2+^ can be observed. And the concentration of Fe^2+^ increases with decreasing the oxygen pressure, which clearly suggests that the oxygen ion concentration in a-YFCO films decreases with decreasing oxygen pressure. As a consequence, there are lots of oxygen-deficiencies in the a-YFCO films and the concentrations of oxygen-deficiencies vary for different oxygen pressures. The presence of oxygen-deficiencies indicates that a CF induced by the interconnected oxygen-deficiencies should be formed or ruptured during the electroforming step and subsequent bipolar RS should take place in a-YFCO films.

**Figure 3:**
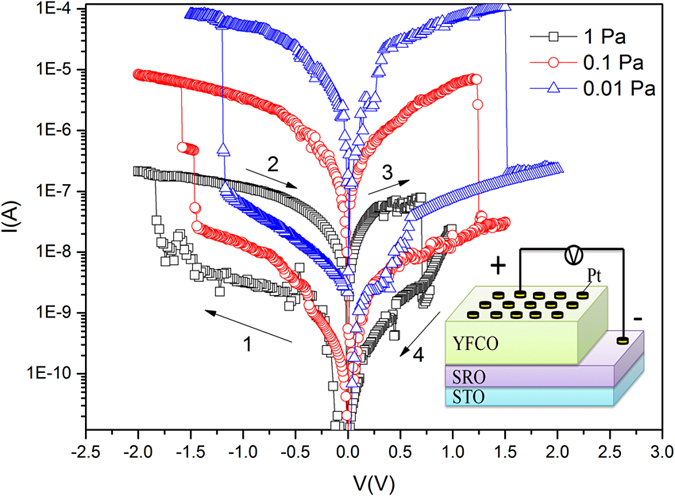
Resistivity switching of Pt/a-YFCO/SRO devices fabricated under different oxygen pressures. Inset shows a schematic of the device structure.

Figure 3 shows the I–V curves of a-YFCO films. A two-terminal method is used for the *I–V* measurement at room temperature, as shown in the inset of Fig. 3. The forward bias is defined by the current flowing from the top electrode (TE) to the bottom electrode (BE), and the negative bias is defined as the opposite. Pt and SRO were the TE and BE, respectively. Bias voltages were applied to the Pt TE when the SRO BE was grounded for all measurements. The voltage bias was scanned as follows: 0 → −V_max_ → 0 → +V_max_ → 0. Hysteresis and asymmetry RS effects were observed. By steadily increasing the negative voltages applied to the devices, a pronounced change of resistance, from HRS to the LRS, was observed, which is referred as the “SET” process. Subsequently, an opposite “RESET” process could be seen when sweeping the voltage back to the positive value. Figure 3 shows that a larger loop in the *I–V* curve could be obtained in a-YFCO films fabricated under 0.01 Pa, indicating that larger RS can be achieved in the a-YFCO films with much more oxygen-deficiencies.

**Figure 4:**
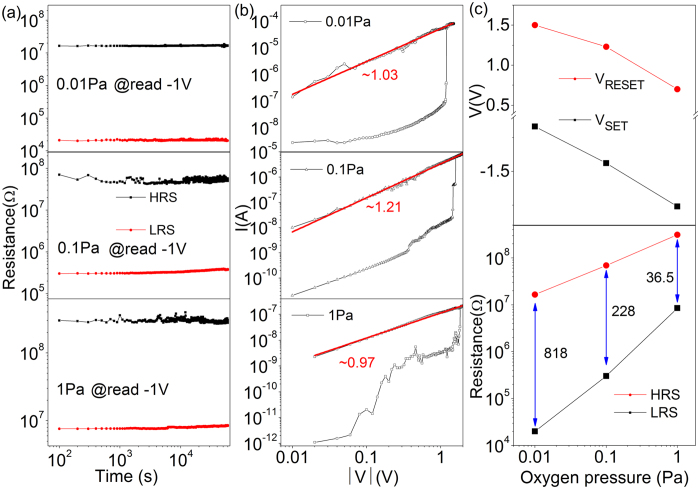
(**a**) Retention performance of the HRS and LRS of the devices. (**b**) Logarithmic plot and linear fitting of the previous *I–V* curve. (**c**) Switching voltages and R_HRS_/R_LRS_ ratios for different devices.

Figure 4(a) shows the retention property of a-YFCO up to 10^4^ s and there is no significant reduction of resistance over the entire range, suggesting that a very stable RS was generated in a-YFCO films. To better understand the conduction and switching mechanism of the memory device, the *I–V* characteristics are plotted on a log-log scale. Figure 4(b) shows the logarithmic plot and linear fitting for the *I–V* curves at the negative voltage sweep region. The *I–V* in LRS clearly shows an Ohmic conduction, which is associated with the formation of CF in device during the SET process^29^,^47^. As shown in the Figure S2 of Supporting Information, the resistance in LRS is independent of the area of electrode, and the resistance in HRS shows a linear dependence on the area of electrode. These results further suggested the filamentary switching mechanism works well in the a-YFCO films^36^,^37^. In fact, the oxygen-deficiency is responsible for the creation of a CF network across the device. When the voltage firstly sweeps from zero toward a positive voltage, the RS hysteresis is also observed. This suggests that RS in a-YFCO is a bipolar RS effect. Therefore, it can be concluded that oxygen-deficiencies significantly affect the RS property of the a-YFCO-based devices. The interconnected or disconnected oxygen-deficiencies at various applied voltages will result in the formation or rupture of the CF in oxide films, respectively^48^,^49^,^50^. Figure 4(c) shows the range of “SET” and “RESET” voltages for different oxygen pressures. Clearly, the “SET” voltage increases with increasing oxygen pressure, which means that the CF was easily formed if there were a large amount of oxygen-deficiencies in the a-YFCO. With decreasing concentration of oxygen-deficiencies, a higher voltage is needed to form the CF. On the other hand, the “RESET” voltage decreases with increasing oxygen pressure, which suggests that the CF induced by the interconnected oxygen-deficiencies can be easily ruptured if there are a small amount of oxygen-deficiencies in the films. In Fig. 4(c), we can also find that the R_HRS_/R_LRS_ ratio, indicated by the numbers, becomes larger with decreasing oxygen pressure. These results suggest that the RS effect becomes more pronounced in films containing much more oxygen-deficiencies. This also confirms that the migration of oxygen-deficiencies play a crucial role in the RS property of the a-YFCO-based device. Furthermore, the RS properties of SRO cannot be completely excluded^8^, and the SRO can be considered as a partial V_O_s reservoir in the formation of a continuous CF between the TE and BE.

**Figure 5:**
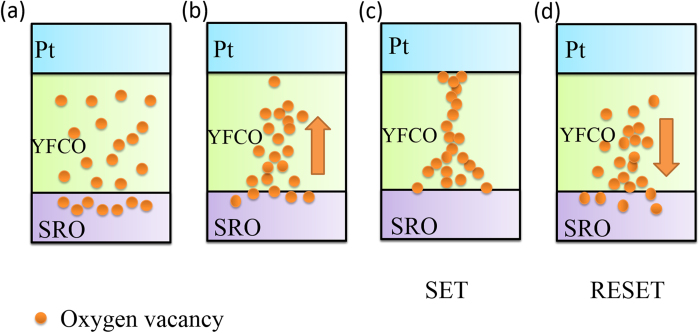
Schematic diagrams of the RS mechanism of the Pt/a-YFCO/SRO device.

Figure 5 shows an illustration of the CF model for the RS mechanism of Pt/a-YFCO/SRO. A dynamic model of oxygen-deficiencies gathering and migrating was proposed to interpret the RS mechanism. There are lots of oxygen-deficiencies in the a-YFCO and SRO films due to their preparation conditions. Oxygen-deficiencies can be driven away by the “SET” electrical stimulus, and then return to the original sites during the “RESET” process. In the HRS, a current probably originates from defects without forming filaments of interconnected oxygen-deficiencies, as shown in Fig. 5(a–d). On the other hand, the filaments will be formed due to the oxygen-deficiencies migration driven by electric field and subsequent LRS was observed, as shown in Fig. 5(c). When a negative voltage is applied, lots of oxygen-deficiencies are driven from BE to TE [Fig. 5(b)]. Once the voltage sweeps to a certain value, oxygen-deficiencies arrange themselves to form a continuous CF between the TE and BE, as shown in Fig. 5(c). The electrons flow along the chain of lower valent cations that are caused by the oxygen-deficiencies. As a result, the system enters the LRS. When a positive voltage is applied to the device during “RESET” process, the interconnected oxygen-deficiencies in the a-YFCO films were ruptured due to the oxygen-deficiencies migrating toward the BE and the system enters the HRS [Fig. 5(d)]. If the voltage sweeps firstly from zero to a positive voltage, the RS hysteresis can also be expected because the migration of oxygen-deficiencies can be tuned by different electric field.

## Conclusions

In summary, we have prepared a series of a-YFCO films under various oxygen pressures using pulsed laser deposition. XRD and TEM patterns clearly confirmed that a-YFCO films were produced. The XPS data verified that the concentration of oxygen-deficiencies increases with decreasing oxygen pressure in a-YFCO films. Larger RS behavior can be obtained in a-YFCO films with much more oxygen-deficiencies at room temperature. The variation of the “SET” (or “RESET”) voltage and the R_HRS_/R_LRS_ ratio indicate that oxygen-deficiencies play vital role in the RS behaviors of a-YFCO. Based on the experimental results, including Ohmic conduction measurements and the resistance independent on the area of electrode at LRS, we can confirm that the formation/rupture of a CF is the origin of RS. It is well known that oxygen-deficiencies in oxide films can be easily manipulated during the fabrication process. The enhanced RS and long retention in a-YFCO suggest it has a great potential for applications in nonvolatile memory devices. This work provides us understandings on the basic mechanism of the RS effects and guidelines for high-performance devices.

## Methods

Sintered polycrystalline ceramic disks of pure YFCO^35^ and SrRuO_3_ (SRO) (99.99%, Alfa Aesar) with a diameter of 10 mm and a thickness of 5 mm were used as target materials. ~20 nm SRO film was grown using pulsed laser deposition (PLD) on (001)-oriented SrTiO_3_ (STO) single-crystal substrates under flowing oxygen at 10 Pa and 650 °C using a KrF excimer laser (λ = 248 nm) with a repetition rate of 5 Hz and a fluence of 200 mJ. The laser spot size and corresponding energy density applied during the growth are about 2 mm^2^ and 100 mJ/mm^2^, respectively. ~20 nm YFCO films were grown at 700 °C with different oxygen pressures to tune the concentrations of oxygen-deficiencies in films. The deposition rate of SRO and a-YFCO is about 2 and 1 nm/min., respectively. Pt top electrodes of 50, 100, 200 μm in diameter and 10 nm in thickness were sputter-deposited with a shadow mask. X-ray diffraction (XRD) data were collected using the X’Pert XRD spectrometer with Ni-filtered Cu *Kα* radiation. X-ray photoelectron spectroscopy (XPS) was measured with an ESCALAB 250Xi (Thermo Fisher Scientific). The high-resolution transmission electron microscopy (HRTEM) measurement is carried out using a JEOL Model 2010 TEM. The resistance and *I–V* curves were measured with a Keithley 2601 power source.

## Additional Information

**How to cite this article**: Wang, X. *et al*. Effect of Oxygen-deficiencies on Resistance Switching in Amorphous YFe_0.5_Cr_0.5_O_3−d_ films. *Sci. Rep*. **6**, 30335; doi: 10.1038/srep30335 (2016).

## Supplementary Material

Supplementary Information
