# Supplementary material for: Effect of Oxygen-deficiencies on Resistance Switching in Amorphous YFe0.5Cr0.5O3−d films
Source: Sci Rep. 2016 Jul 25;6:30335. doi: 10.1038/srep30335 (PMC4959013; doi:10.1038/srep30335)
Supplement: Supplementary Information [file srep30335-s1.pdf]

Supporting Information

Effect of Oxygen-deficiencies on Resistance Switching in Amorphous $YFe_{0.5}Cr_{0.5}O_{3-d}$ films

Xianjie Wang[1, a)], Chang Hu[1], Yongli Song[1], Xiaofeng Zhao[1], Lingli Zhang[1], Zhe Lv[1], Yang Wang[1,2], Zhiguo Liu[1], Yi Wang[1,2], Yu Zhang[1], Yu Sui[1, a)] and Bo Song[1,2 , a)]

[1] Department of Physics, Harbin Institute of Technology, Harbin 150001, China

[2] Academy of Fundamental and Interdisciplinary Sciences, Harbin Institute of Technology, Harbin 150001, China

Table S1. ICP analysis of a-YFCO film

	Y	Fe	Cr
Analytical wavelength (nm)	324.227	238.204	267.716
Atomic weight	88.90585	55.845	51.9961
Experimental data(mg/L)	1.384	0.435	0.405

The **a-YFCO film on STO substrate** was dissolved in 1 L solution with 100 ml 10% nitric and 100 ml 20% hydrochloric acid. The solution is diluted ten times again to take the ICP test (PerkinElmer, ICP-OES 5300DV). In accordance with the calculation, the mole ratio of Y, Fe and Cr is 2:1:1 with the consistent design.

Author to whom correspondence should be addressed; Email: wangxianjie@hit.edu.cn (X. Wang); suiyu@hit.edu.cn (Y. Sui): songbo@hit.edu.cn (B. Song)

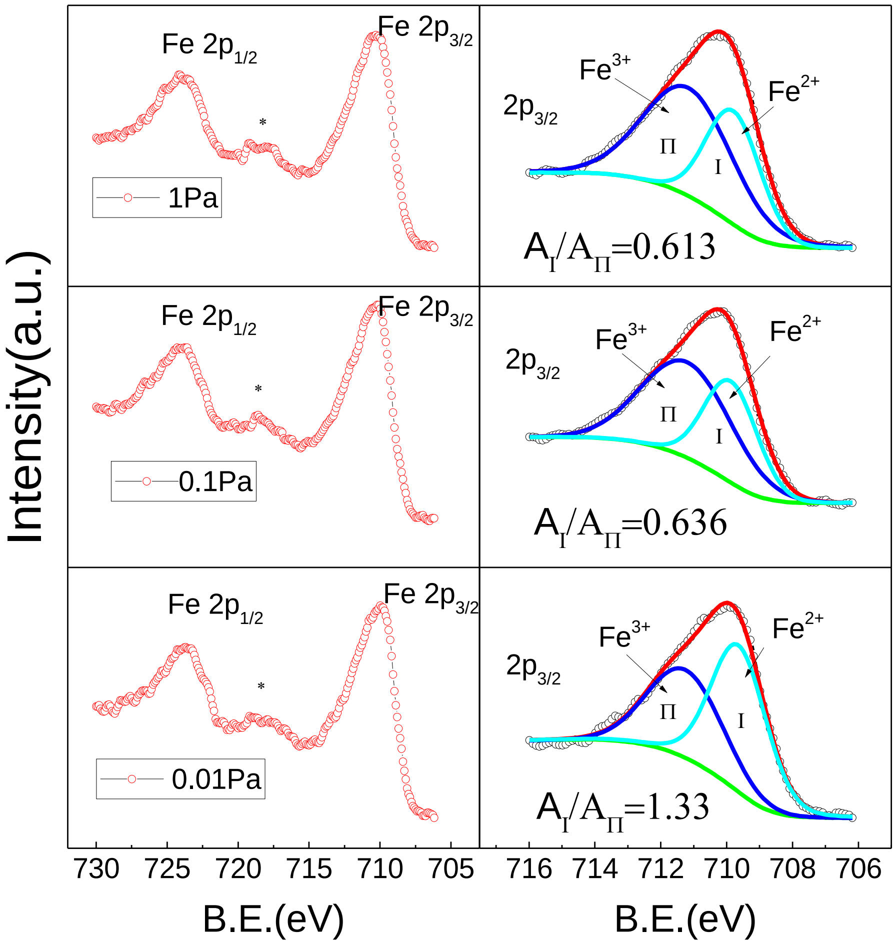

Fig S1. The Fe 2p XPS data in the a-YFCO films. Clear signals of Fe^{3+} and Fe^{2+} can be observed. And the concentration of Fe^{2+} increases with decreasing the oxygen pressure, which clearly suggests that the oxygen ion concentration in a-YFCO films decreases with decreasing oxygen pressure.

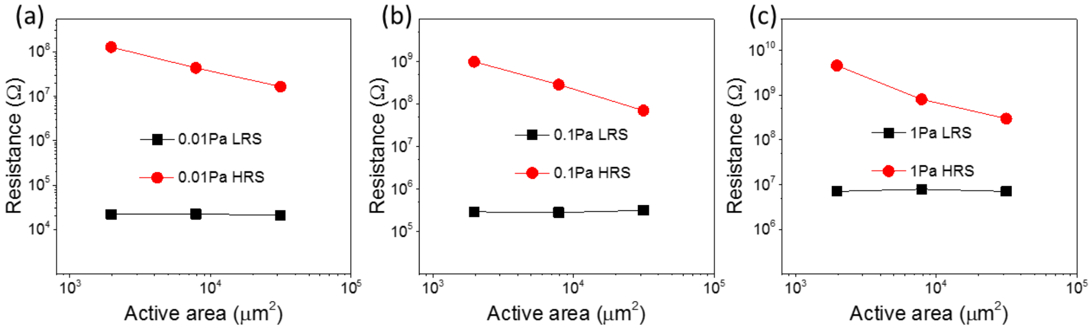

Fig S2. The area-dependence of the resistance upon switching. The resistance in LRS is independent of the area of electrode, and the resistance in HRS shows a linear dependence on the area of electrode. These results suggested the filamentary switching mechanism works well in the a-YFCO films.[S1,S2] In fact, the oxygen-deficiency is responsible for the creation of a filament network across the device.

S1. Yoo, S., Eom, T., Gwon, T. & Hwang, C. S. Bipolar resistive switching behavior of an amorphous $Ge_2Sb_2Te_5$ thin films with a Te layer. *Nanoscale* **7**, 6340-6347 (2015).

S2. Waser, R., Dittmann, R., Staikov, G. & Szot, K. Redox-Based Resistive Switching Memories - Nanoionic Mechanisms, Prospects, and Challenges. *Adv. Mater.* **21**, 2632-2663 (2009).
